# The Association of *Toxoplasma gondii* IgG Antibody and Chronic Kidney Disease Biomarkers

**DOI:** 10.3390/microorganisms10010115

**Published:** 2022-01-06

**Authors:** Amani Babekir, Sayed Mostafa, Emmanuel Obeng-Gyasi

**Affiliations:** 1 Department of Built Environment, North Carolina A&T State University, Greensboro, NC 27411, USA; aebabekir@aggies.ncat.edu; 2 Environmental Health and Disease Laboratory, North Carolina A&T State University, Greensboro, NC 27411, USA; 3 Department of Mathematics and Statistics, North Carolina A&T State University, Greensboro, NC 27411, USA; sabdelmegeed@ncat.edu

**Keywords:** toxoplasma, kidney disease, renal disease, *T. gondii*

## Abstract

Background: *Toxoplasma gondii* (*T. gondii*) is a parasite that infects more than 40 million Americans and causes toxoplasmosis. Most cases of toxoplasmosis are asymptomatic; however, *T. gondii* is capable of invading organs like the kidney, causing chronic infections and cell destruction. Methods: This study focused on evaluating the association between *T. gondii* exposure and chronic kidney disease (CKD) using data from the 2009–2010 National Health and Nutrition Examination Survey (NHANES). *T. gondii* exposure was assessed using *Toxoplasma gondii* IgG antibody status, and the status of CKD was assessed using the CKD biomarkers. The evaluation of risk rate and population prevalence was performed. In addition, multivariable regression models were used to further investigate this association after adjusting for sociodemographic, anthropometric, behavioral, and clinical covariates commonly associated with kidney dysfunction. Results: The positive *T. gondii* IgG antibody participants had significantly higher levels of CKD biomarkers, including second albumin-to-creatinine ratio (*p* = 0.0376), second albuminuria (*p* = 0.0005), and persistent albuminuria (*p* < 0.0001) compared to the negative participants. Furthermore, there were statistical associations between *T. gondii* exposure and the status of CKD (negative vs. positive) (*p* = 0.0001), and between *T. gondii* exposure and the CKD stage (negative, stage 1, …, stage 5) (*p* = 0.0004). Without adjusting for age, the positive *T. gondii* participants had a significantly higher risk (27% higher) of having CKD than the negative participants (RRcrude = 1.27, 95% CI: 1.09–1.49). The age-adjusted prevalence of CKD was higher among Toxoplasma-positive participants compared to the Toxoplasma-negative participants (10.45 vs. 8.99). *T. gondii* infection was significantly associated with CKD (OR = 1.40, 95% CI = 1.06–1.84, *p* = 0.00447) after adjusting for age, gender, race/ethnicity, and BMI. Age was positively associated with CKD (OR = 8.89, 95% CI = 6.31–12.51, *p* < 0.0001) with the participants 45+ years old being 8.89 times more likely to have CKD than those who are <45 years old, after adjusting for *T. gondii* infection, gender, race/ethnicity, and BMI. Moreover, positive *T. gondii* increased the odds of CKD progression (OR = 1.41, 95% CI = 1.07–1.86, *p* = 0.0424). Conclusions: Positive *T. gondii* IgG antibody is associated with CKD and the progression of CKD stages. This association is more apparent among older people. Further investigations are needed to examine these findings in different geographical locations and among differentially exposed populations.

## 1. Introduction

### 1.1. Toxoplasma

*Toxoplasma gondii* (*T. gondii*) is a protozoan parasite that has a worldwide geographic distribution; it infects more than 40 million individuals in the United States of America (USA), with high prevalence among Mexican Americans and other-Hispanics as compared to the other racial/ethnic groups [1]. *T. gondii* infection causes toxoplasmosis, the leading cause of death related to foodborne illnesses in the USA [2]. Toxoplasmosis, in most cases, has no symptoms; however, chronic infection develops when the parasite invades the organs and destroys tissues forming cysts [3].

*T. gondii* exists in three infectious forms: tachyzoite (rapid reproducing form), bradyzoite (inside tissue cyst), and sporozoite (inside oocysts) [3,4]. Moreover, its life cycle includes mammals and birds as intermediate hosts, while felines are the main hosts. Cats get infected with *Toxoplasma* by eating small animal tissues containing *T. gondii* cysts; then the cyst walls dissolve in the stomach releasing bradyzoites, which multiply in the small intestine wall and form oocysts. After 3–14 days of infection, cats can shed millions of oocysts in their feces [5]. The excreted oocysts sporulate within 1–5 days surviving environmental conditions and contaminating the surroundings and water. Therefore, the intermediate host contracts *T. gondii* by consuming contaminated food or water, and the parasite is capable of surviving in their tissues for several years [6]. In addition, humans may get infected through congenital transmission or organ transplants such as kidney transplants [7]. Inside the human body, bradyzoites form hundreds of tachyzoites, which circulate in the bloodstream and invade organs such as the brain, heart, liver, and kidney, causing inflammation and damage [8].

The susceptibility to infection and pathogenicity of *T. gondii* depends on the strain, immune system status, and genetic factors. *T. gondii* strains have several genotype groups, including type I, II, and III, which are different in geographical distribution and virulency, with strain type II being the most common strain in North America [9]. Regardless of the strain group, the human immune system usually prevents toxoplasmosis pathogenicity with the exception of pregnant women and immunocompromised individuals. When pregnant women get infected with the parasite, the tachyzoites may cross the placenta and infect the developing fetus causing diseases, growth failure, or abnormalities [10]. Furthermore, the pathogenicity of toxoplasmosis is linked to an increase in oxidative stress and inflammation; thus, it is associated with various diseases such as heart diseases, obsessive-compulsive disorder, and schizophrenia [11,12,13]. Moreover, Babekir et al. [14] indicated that *T. gondii* seropositivity is associated with elevated levels of biomarkers and clinical markers such as triglycerides (TG), systolic blood pressure (SBP), low-density lipoprotein cholesterol (LDL), gamma-glutamyl transferase (GGT), and C-reactive protein (CRP).

The clinical diagnosis of toxoplasmosis includes serological tests detecting *T. gondii* IgG and IgM antibodies. The IgG antibody test is used to detect acute or chronic phases, while the IgM antibody test is used to confirm acute phases [15].

### 1.2. Chronic Kidney Disease

Chronic Kidney Disease (CKD) is the gradual decrease in the kidney’s capability to filter blood [16]. It is estimated that 37 million individuals in the USA have CKD; approximately 90% of them are not aware they have the disease [17]. The major issue with CKD is that the progressive loss of kidney function is irreversible, and the typical symptoms of kidney dysfunction appear in the late stage of the disease [18]. The progression of CKD leads to the accumulation of toxic waste in the blood, causing severe health complications such as high blood pressure, heart disease, stroke, and mortality [17]. The accumulation of toxic waste increases reactive oxygen species production and contributes to tissue damage; furthermore, the deterioration in kidney function weakens the immune response and increases the vulnerability to infections [19].

Identifying the individuals with an increased risk of CKD will enable early interventions and reduce the burden of the disease. Therefore, several risk factors are recognized, including genotype, age, gender, race, and family history [20]. CKD is more common among the elderly, women, and non-Hispanic Black adults [18]. In addition, obesity, smoking, and uncontrolled diabetes or hypertension are associated with CKD; moreover, acute kidney injury, cardiovascular diseases, and analgesic medication constitute risks [20].

The common biomarkers used to detect kidney dysfunction are albumin and creatinine. Albumin is a protein that circulates in the blood with a well-functioning kidney preventing albumin from getting excreted in the urine. Thus, the level of albumin in the urine (albuminuria) is used as an indicator of kidney health [21]. Creatinine is a waste product of metabolism in the muscle released to the blood—a healthy kidney is able to filter creatinine from the blood [22].

CKD is diagnosed by the presence of glomerular filtration rate (GFR) lower than 60 mL/min/1.73 m^2^ for at least three months or the presence of GFR greater than 60 mL/min/1.73 m^2^ along with evidence of kidney injury [23]. The common indicators of kidney injury are albuminuria, proteinuria, and an abnormal MRI kidney image. Albuminuria is indicated by the level of albumin in the 24 h urine (>30 mg) or the level of albumin in the urine sample adjusted by urinary creatinine (>30 mg/g). Accordingly, albuminuria is categorized to three stages: normal (<30 mg/g), moderate (30–300 mg/g), and severe (>300 mg/g) [23].

CKD progresses in stages; in the early stages, the kidneys are still capable of filtering out waste, while in the latter stages, the kidneys have lost most of their capabilities or stopped working. Thus, CKD has five stages: stage 1 (mild kidney damage, eGFR ≥ 90), stage 2 (mild kidney damage, eGFR 60–89), stage 3 (moderate kidney damage, 30 and 59), stage 4 (moderate/severe kidney damage, eGFR 15–29), and stage 5 (kidney failure, eGFR ≤ 15) [24].

The NHANES survey incorporates the surveillance of renal disease among the USA population by testing renal and kidney biomarkers and defining four stages of CKD using estimated GFR (eGFR) and estimated persistent albuminuria; the persistent albuminuria is used to determine stage 1 and stage 2 CKD [25].

The NHANES data reveal that the prevalence of CKD among the USA population is in a stable trend and is associated with obesity and elevated FG, TG, TC, and HDL [26,27]. The accumulative data from NHANES 2003–18 indicated that the prevalence of CKD among different races was 20% (non-Hispanic white), 19% (non-Hispanic black), 15% (Hispanic), and 14% (other) [27]. However, there is an increasing trend of CKD among Mexican Americans, and there is a need to further investigate the risk factors associated with these groups [28].

### 1.3. Toxoplasma and CKD 

Previous studies indicated that patients undergoing dialysis have a higher rate of active *Toxoplasma* infection compared to healthy individuals. This could be due to the weakness of the immune system and the reactivation of a chronic *Toxoplasma* infection [29]. In addition, *T. gondii* seropositive pregnant women had significantly higher serum creatinine and blood urea than the seronegative group [30]. On the other hand, CKD may reduce the immune response to *T. gondii* exposure and facilitate the infection with toxoplasmosis [31,32]. Therefore, the purpose of this study is to further investigate the association between *T. gondii* and CKD and explore the significance of this association among the different demographic groups.

## 2. Materials and Methods

### 2.1. Study Population

This study’s sample was a sub-sample of the population in the NHANES 2009–2010 who were 20 years or older and tested for *T. gondii*. The NHANES 2009–2010 is a multistage stratified survey designed to provide a detailed examination of the health and nutritional status of a nationally representative sample of non-institutionalized individuals in the USA. The National Center for Health Statistics of the Centers for Disease Control and Prevention Institutional Review Board approved the protocols for NHANES 2009–2010, and all participants gave informed consent. The sampling process included sampling one county from each of 15 groups of counties, then sampling segments, households, and persons from the selected counties. The demographic information was collected through a computer-assisted personal interview (CAPI).

### 2.2. Exposure Variable: T. gondii 

The antibodies of *T. gondii* were analyzed with specific Toxoplasma IgG enzyme immunoassay kits (Bio-Rad, Redmond, WA, USA). Results were reported as IU/mL and coded as positive (≥33 IU/mL) or negative (<27 IU/mL). Samples with equivocal results (≥27 IU/mL and <33 IU/mL) were repeated twice and confirmed as negative.

### 2.3. CKD biomarkers

#### 2.3.1. First and Second Albumin-to-Creatinine Ratios

The first albumin creatinine ratio (mg/g) was calculated by dividing the random (first collection) urine albumin by the first urine creatinine collection. The second albumin creatinine ratio (mg/g) was calculated by dividing the second collection of albumin by the second urine creatinine.

#### 2.3.2. First and Second Albuminuria

Albuminuria was categorized using the calculated albumin-to-creatinine ratios: microalbuminuria (30–299 mg/g) and macroalbuminuria (>300 mg/g).

#### 2.3.3. Persistent Albuminuria

Persistent albuminuria was defined by having albuminuria in both first and second urine samples.

#### 2.3.4. Serum Creatinine (Scr)

A standardized method was used to measure creatinine in the blood. A volume of sample was introduced into a reaction cup containing an alkaline picrate solution. Absorbance readings were taken at 520 nm between 19 and 25 s after sample injection. The absorbance rate has been shown to be a direct measure of the concentration of creatinine in the sample.

#### 2.3.5. eGFR

The eGFR was estimated using the formula recommended by NHANES (MDRD study formula):

eGFR (mL/min/1.73 m²) = 175 × (Scr)^−1.154^ × (Age)^−0.203^ × (0.742 if female) × (1.210 if African American)

eGFR was classified into five stages based on the ranges of eGFR used in the CKD classification:

Stage 1: eGFR ≥ 90 mL/min/1.73 m^2^;

Stage 2: eGFR 60–89 mL/min/1.73 m^2^;

Stage 3: eGFR 30–59 mL/min/1.73 m^2^;

Stage 4: eGFR 15–29 mL/min/1.73 m^2^;

Stage 5: eGFR < 15 mL/min/1.73 m^2^.

#### 2.3.6. CKD

CKD was classified into negative and positive, with positive representing all the stages of CKD. The CKD stages include:

- Stage 1: eGFR ≥ 90 mL/min/1.73 m^2^ and estimated persistent albuminuria;
- Stage 2: eGFR 60–89 mL/min/1.73 m^2^ and estimated persistent albuminuria;
- Stage 3: eGFR 30–59 mL/min/1.73 m^2^;
- Stage 4: eGFR 15–29 mL/min/1.73 m^2^;
- Stage 5: eGFR < 15mL/min/1.73 m^2^.

Furthermore, CKD was classified into three levels [33]:

- Negative: Negative;
- Mild CKD: Stage 1 and 2;
- Moderate-to-Severe CKD: Stage 3, 4, and 5.

### 2.4. Covariates for Models Adjustment

The variables used for model adjustment included diabetes, BMI, gender, age, race/ethnicity, alcohol use, smoking, and drug use. These are the most common risk factors associated with CKD [27].

### 2.5. Statistical Analysis

The statistical analysis was conducted using the *Survey* package in R (version 4.0.2; R Foundation for Statistical Computing, Vienna, Austria), considering the survey weights and the sampling design of NHANES. The evaluation of the association between *T. gondii* IgG and kidney biomarkers included Rho-Scott chi-square bivariate analyses, design-based t-tests, and logistic regression. Stratification and the Mantel-Haenszel pooled risk ratio estimate were used to investigate the confounding effect of age. A *p*-value < 0.05 was considered significant in all of these analyses. The regression models were adjusted for demographic, behavioral, and anthropometric covariates.

## 3. Results

### 3.1. Data Summary

A descriptive summary (survey-weighted percentages or means and standard errors) of the variables used in the analysis are presented in Table 1. The total number of sampled participants used in this analysis was 4692, of whom 47.7% were males, and 52.3% were females. The mean age was 47.9 years, and the percentages of Mexican American, other Hispanic, non-Hispanic white, non-Hispanic black, and other race were 8.4, 4.8, 70.3, 9.9, and 6.5, respectively. The percentage of *T. gondii* IgG-positive participants was 15.2%, while the percentage of participants with albuminuria was 6.9% (first) and 4.0% (second). The percentage of participants with persistent albuminuria was 3.5%, and the average of eGFR was 87.8 mL/min/1.73 m^2^. The percentage of CKD-positive participants was 10%, including 0.9% in stage 1, 1.5% in stage 2, 7.1% in stage 3, and 0.5% in stage 4. The portion of participants with mild CKD was 2.4%, and those with moderate-to-severe CKD was 7.6%.

### 3.2. Association of Toxoplasma and CKD Biomarkers and CKD Status

**Figure 1:**
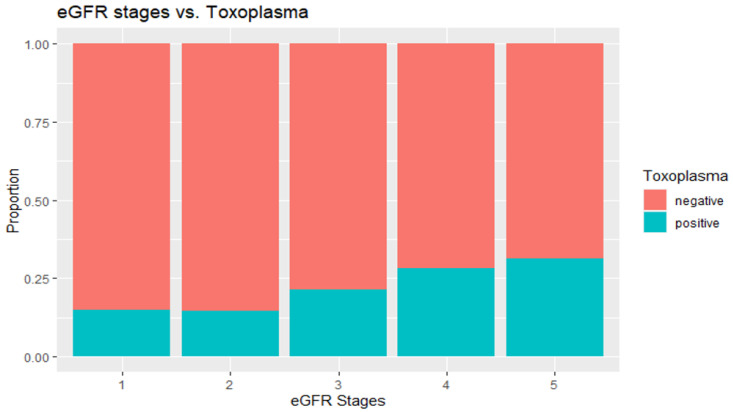
eGFR levels by *T. gondii* exposure status.

Table 2 presents the associations between *T. gondii* IgG status and CKD biomarkers without accounting for any covariates. The design-based *t*-test or Rho-Scott chi-square was used to assess the significance of these associations. The results indicated that there was statistically significant associations between the *T. gondii* status and each of second albumin-to-creatinine ratio (*p* = 0.0376), second albuminuria (*p* = 0.0005), persistent albuminuria (*p* < 0.0001), CKD status (*p* = 0.0001), and CKD stages (*p* = 0.0004). The highest proportion of *T. gondii*-positive patients occurred in stage 4, while the mean of eGFR was lower among the *T. gondii*-positive participants, but it was not statistically significant (*p* = 0.0913). However, the level of eGFR was significantly different among positive and negative participants (*p* = 0.0014). The elevated level of eGFR (stages 4 and 5) had higher proportions of positive participants than the negative participants (Figure 1). Furthermore, there was a statistically significant association between the *T. gondii* status and CKD levels with higher proportions of mild and moderate-to-severe levels of CKD among the positive participants when compared to the negative participants (*p* < 0.0001).

### 3.3. Age Factor

**Figure 2:**
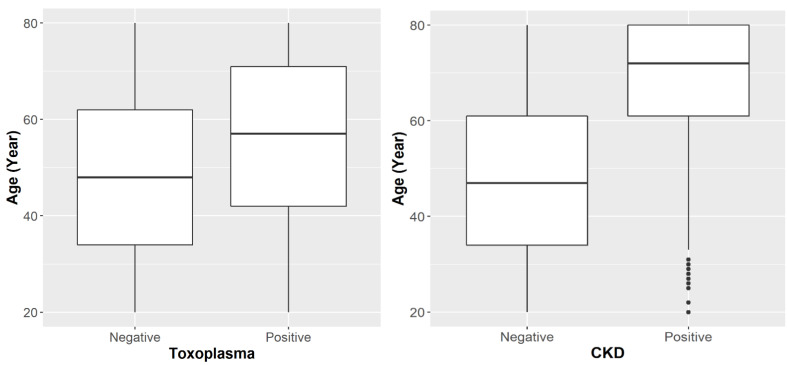
Age distribution by *T. gondii* exposure status and CKD status.

Figure 2 shows that the participants who were exposed to *Toxoplasma* were significantly older than the non-exposed participants (56 years vs. 49 years, *p*-value < 0.0001). Similarly, the participants with positive CKD were significantly older than the negative participants (67 years vs. 48 years, *p*-value < 0.0001). We investigated the possibility of confounding effect for age on the association between *T. gondii* exposure and CKD status. The magnitude of the confounding effect of age was evaluated by calculating the CKD risk ratio within each age stratum (younger, older) and comparing it with the unstratified risk ratio (Table 3). The unstratified risk ratio of CKD (RR_crude_ = 1.27, 95% CI: 1.09–1.49) indicated that participants with positive *Toxoplasma* had a significantly higher (27% higher) risk of having CKD than the negative participants. While the stratified risk ratio was higher among the older participants than the younger participants (1.11 vs. 0.76), the risk ratio was insignificant in both age groups, as indicated by the corresponding 95% confidence intervals. The unstratified and stratified effect estimates differ by 17%, indicating the magnitude of the confounding factor of age. The overall risk ratio was calculated by pooling the stratified risk ratios using the Mantel-Haenszel formula [34] (RR_MH_ = 1.09, 95% CI: 0.93–1.28), which indicated an insignificant difference between the positive and negative *T. gondii* participants. These results clearly indicate that age is an important confounding factor that must be accounted for when studying the association between *T. gondii* exposure and CKD. To account for other potential covariates in addition to age, we made use of multivariable logistic regression models as detailed below.

**Figure 3:**
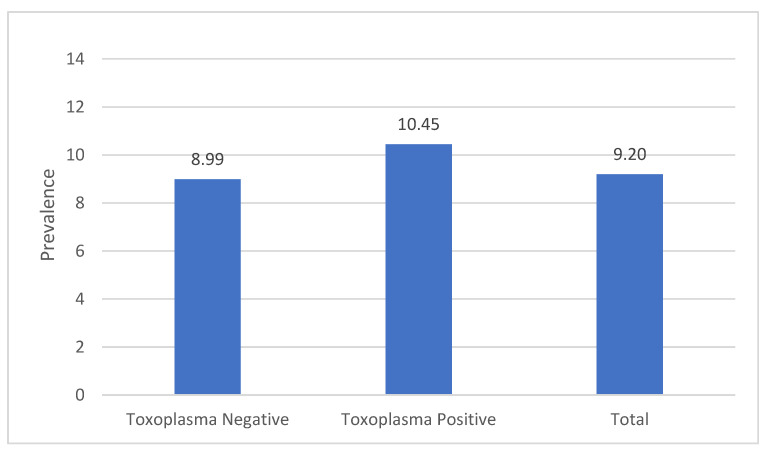
Age-adjusted prevalence of CKD by *T. gondii* exposure status. Total = *T. gondii*-positive and negative participants.

The age standardization method recommended by NHANES was used to remove the confounding effect of age when comparing the prevalence of CKD by *Toxoplasma* in the USA population. The age distribution was standardized using the 2000 US census population. Figure 3 shows that the prevalence of CKD was higher among *Toxoplasma* positive than *Toxoplasma* negative individuals (10.45 vs. 8.99).

### 3.4. Association of Toxoplasma and CKD after Adjusting for Co-Variates

Several binomial logistic regression models were created to investigate the association between CKD status (negative vs. positive) and *Toxoplasma* exposure (negative vs. positive) while accounting for potential covariates. The first model describes the association between CKD status and *Toxoplasma* exposure adjusted for demographic covariates (age, gender, and race/ethnicity) and BMI. Table 4 summarizes the results of this first model. In this model, *T. gondii* was significantly associated with CKD (OR = 1.40, 95% CI = 1.06–1.84, *p*-value = 0.00447). Specifically, positive *T. gondii* significantly increased the odds of having CKD, holding all other variables unchanged. Age was also positively associated with CKD (OR = 8.89, 95% CI = 6.31–12.51, *p*-value < 0.0001), with the participants 45+ years old being 8.89 times more likely to have CKD than those who are <45 years old. Additionally, the model showed that female participants were more likely to have CKD (27% higher odds than males, *p*-value = 0.0352), whereas there were no significant differences among the different race/ethnicity groups. BMI had slight positive association with CKD (OR = 1.04, 95% CI = 1.02–1.06, *p*-value = 0.0059).

In the second model, additional covariates were added, including alcohol use and diabetes status (Table 5). The effect of *T. gondii* was not significant in this model; however, age continued to have a strong association with CKD status (OR = 8.59, 95% CI = 5.48–13.47, *p*-value = 0.0002). Unlike the first model, gender did not have any significant association with CKD status in the second model. Race/ethnicity did not show any significant association with CKD status, which is similar to the conclusion obtained from the first model. Furthermore, participants with no history of diabetes had 60% lower odds of having CKD (OR = 0.40, 95% CI = 0.27–0.61, *p*-value = 0.0075) than those with a history of diabetes. Alcohol use did not have a significant association with CKD status (*p*-value = 0.7102). The model in Table 5 provides a somewhat better fit for the data than the model in Table 4, as indicated by the better fit statistics (lower AIC and higher Pseudo R-Square for the model in Table 5).

A third model was created for CKD status on *T. gondii* IgG antibody by dropping age from the second model but keeping all other covariates. This model showed a significant association between *T. gondii* exposure and CKD status (OR = 1.56, 95% CI = 1.20–2.04, *p*-value = 0.0168). However, the second model (model in Table 5) fit the data better than the third model (Table 6), as indicated by the AIC and R-Square values for the two models.

Additional variables, including smoking and drug use, were added to the third model to investigate the association of Toxoplasma and CKD status in the presence of these conditions (Table 7 and Table 8). The association of Toxoplasma was significant in the CKD status when the smoking risk was considered (*p* = 0.0325); however, the addition of drug use variables reduced the sample size to 1551 and defused the effect of Toxoplasma and the other variables. 

To investigate the association between the progression in the CKD stages and *Toxoplasma* while adjusting for potential covariates, we built the ordinal logistic regression model described in Table 9. The model showed a significant association between *T. gondii* exposure and CKD stages (OR = 1.41, 95% CI = 1.07–1.86, *p*-value = 0.0424), which indicates that positive *T. gondii* increases the odds of CKD progression. In this model, age, gender, and BMI were all significantly associated with CKD progression, but race/ethnicity was not.

Additional models were created to investigate the association of Toxoplasma and CKD using two levels of CKD (mild and moderate-severe). The association of Toxoplasma was not apparent when the age factor was included in the model (Table 10). Nevertheless, excluding age from the model (Table 11) showed a significant association between levels of CKD and Toxoplasma (*p* = 0.0371)

## 4. Discussion

### 4.1. Overview of Results and Implications

The Centers for Disease Control and Prevention (CDC) estimates that 15% of US adults have CKD, with 90% of these individuals unaware of their condition [17]. Therefore, it is critical to investigate the risk factors associated with CKD to monitor and diagnose it at early stages adequately. 

Exposure to *T. gondii* may cause acute or chronic damage to the kidney, triggering injury, which can affect the exposure over their life course. Prior studies have found a link between undergoing dialysis and an increased rate of *T. gondii* infection [35,36], but there has not been a thorough investigation of the association of *Toxoplasma* with CKD in the USA population. Thus, the purpose of this study was to investigate this association along with common risk factors of CKD.

The investigation began by examining the level of each individual CKD biomarker among *Toxoplasma*-positive and negative participants. We found that the levels of the second albumin-to-creatinine ratio, the second albuminuria, and persistent albuminuria were significantly higher among the *Toxoplasma*-positive participants when compared with the *T. gondii*-negative study participants (Table 2). These biomarkers are the common indicators of kidney injury and CKD progression [23]. Other studies have linked *Toxoplasma* infection to high levels of serum creatinine and blood urea nitrogen [30], and the results of this study confirmed that adverse levels of CKD biomarkers are associated with *Toxoplasma* infection. Despite the finding that the level of estimated GFR was not significantly lower among the *Toxoplasma*-positive compared to the negative participants, the proportions of elevated levels of eGFR were significantly higher among the positive participants compared to the negative participants (*p* = 0014, Table 2 and Figure 1).

In addition, the *Toxoplasma*-positive participants had a significantly higher proportion of CKD (*p* = 0.0001, Table 2) when both eGFR and persistent albuminuria were used to assess CKD.

Moreover, progression from mild to moderate-to-severe stages of CKD was associated with *Toxoplasma* (*p* < 0.0001, Table 2), and progression to each stage of CKD was associated with *Toxoplasma* infection in this study (*p* = 0.0004, Table 2).

In this study, the likelihood of having CKD was significantly higher with exposure to *Toxoplasma* infection than without exposure (RR = 1.27 95% CI: 1.09–1.49, Table 3). The relative risk is greater than one, an indication that CKD is more likely to occur with *T. gondii* infection.

Prior CKD and *Toxoplasma* studies in the USA population suggest that the prevalence of CKD or *Toxoplasma* is associated with age [1,27]. In this study, the participants with positive *Toxoplasma* or CKD were significantly older than the negative participants (*p* < 0.0001, Figure 2). This significant association between age and each of CKD and *Toxoplasma* indicated that age might serve as a confounding factor in the association between CKD and *Toxoplasma*. This observation was supported by the age-stratified analysis. After age adjustments, the prevalence of CKD was higher among *Toxoplasma*-positive participants compared to the *Toxoplasma*-negative participants (10.45 vs. 8.99, Figure 3).

The association between *Toxoplasma* and CKD was further investigated using multivariable models to account for the multiple potential risk factors associated with CKD. The most common risk factors suggested by previous studies were age, gender, race/ethnicity, and BMI. Our analysis confirmed the association between *Toxoplasma* and CKD after adjusting for these factors, where *Toxoplasma* infection was found to be associated with significantly increased odds of having CKD (OR = 1.40, 95% CI = 1.06–1.84, *p* = 0.0447, Table 4). Additionally, age was significantly associated with CKD status, where older individuals were more likely to have CKD (OR = 8.59, 95% CI = 5.48–13.47, *p* = 0.0002, Table 4).

Prior studies suggested that behavioral factors such as alcohol use, smoking, drug use, and medical history (e.g., having diabetes) contribute to the risk of CKD. Thus, these factors were included in the predictive model to investigate the association of *T. gondii* and CKD. After adding these factors to the model, the number of samples decreased due to the missing values in these variables, and the association of *T. gondii* was not apparent. However, the association of *Toxoplasma* in this model was obvious when the strong effect of age factor was excluded from the model (Table 5, Table 6, Table 7 and Table 8). 

In addition, the results of this study showed that positive *Toxoplasma* increased the odds of progression to more severe stages of CKD (OR = 1.41, 95% CI = 1.07–1.86, *p* = 0.0424, Table 9) after adjusting for the other common factors (age, gender, race/ethnicity, and BMI).

Additional classification of CKD was considered in investigating the *Toxoplasma* association by categorizing CKD into two levels: mild and moderate-to-severe CKD. The predictive model created with this CKD classification did not show the significant association of Toxoplasma; however, the association was obvious after excluding age from the model (OR = 1.42, 95% CI = 1.07–1.90, *p* = 0.0371, Table 11).

Our study confirms there is an association between *T. gondii* infection and CKD. This association could have two directions: (1) infection with CKD increases the risk of *T. gondii* infection, or (2) *T. gondii* infection increases the risk of CKD. Prior studies suggest that CKD reduces the immune response to *T. gondii* exposure or reactivates latent *Toxoplasma* in the body [29,31]. On the other hand, latent *T. gondii* infection could injure and damage kidney tissues [8]. 

The mechanism by which *T. gondii* exposure damages the renal system is unclear. Previous studies have demonstrated that *Toxoplasma* infection leads to an increase in the production of nitric oxide and reactive oxygen species (ROS) in cells, resulting in oxidative stress [37]. This oxidative stress is linked to renal failure and triggers an initial inflammatory response mediated by proinflammatory mediators TNF-alpha and IL-1b, and a transcriptional factor, NF-κB. The later stage of inflammation induces an increase in TGF-beta production, leading to the synthesis of extracellular matrix [38]. Thus, the chronic effect of oxidative stress on kidney tissues is mediated through inflammation, subsequent tissue damage, leading to eventual organ dysfunction [38]. This process must be understood in the context of the markers of interest in this study albumin, creatinine, and eGFR. Kidney dysfunction is measured by the level of albumin and creatinine in the blood—a healthy kidney maintains the level of albumin in the blood and filters creatinine waste from the blood. Thus, CKD biomarkers investigated in this study, including albuminuria and eGFR, give an insight into the association of *Toxoplasma gondii* and CKD. Studies by Gupta et al. have indicated that biomarkers of inflammation are inversely associated with measures of kidney function and positively with albuminuria, with inflammation being more elevated among those with lower eGFR and higher urine albumin to creatinine ratio [39].

### 4.2. Implications of Findings

It was critical to explore the association between *T. gondii* and CKD, as parasitic infection effects on renal diseases are understudied. This was especially critical since research is moving towards understanding the exposome, that is, the entirety of exposures that bring about adverse health outcomes. Examining the effects of *T. gondii* helps to close the gap in the literature on how *T. gondii* and similar apicomplexan parasites contribute to adverse renal health outcomes in the short term and over the life course. With this understanding, future studies will be better able to quantify the effects of *T. gondii* exposure in the context of other exposures, such as environmental and social.

### 4.3. Limitation

While this study focused on establishing the association between *Toxoplasma* infection and CKD, further studies are needed to investigate the direction of this association. Simply put, owing to the cross-sectional design of the study, temporality cannot be determined. Therefore, a longitudinal study would offer better insight into the direction of the exposure and outcomes. The used data included a limited set of biomarkers; additional biomarkers such as cystatin C, β-trace protein (BTP), and Neutrophil gelatinase-associated lipocalin (NGAL) should be considered in future studies.

In addition, a larger sample size would allow for more analysis of subsamples within this study. Thus, caution should be taken when interpreting the results.

## 5. Conclusions

Positive *T. gondii* IgG antibody is associated with CKD and the progression of CKD stages. This association is more apparent among older people. Further investigations are needed to examine these findings in different geographical locations and among differentially exposed populations.

## Figures and Tables

**Table 1:** Summary statistics for the variables included in the analysis (*n* = 4692).

Variables	Description	*n*	Weighted Percentage/Mean (SE)
*T. gondii* IgG antibody	<33 IU/mL (negative)	3789	84.8
	≥33 IU/mL (positive)	903	15.2
Gender	Male	2256	47.7
	Female	2436	52.3
Age		4692	47.9 (0.49)
Age group	Younger (<45)	1792	41.9
	Older (≥45)	2900	58.1
Race/ethnicity	Mexican American	874	8.4
	Other Hispanic	483	4.8
	Non-Hispanic White	2337	70.4
	Non-Hispanic Black	754	9.9
	Other Race	244	6.5
Alcohol use	Yes	694	15.6
	No	3046	84.4
Smoking use	Every day	767	35.9
	Some days	169	6.0
	Not at all	1211	58.1
Marijuana/hashish use	Yes	1440	58.0
	No	1309	42.0
Cocaine/heroin/methamphetamine use	Yes	611	18.5
	No	2824	81.5
BMI		4659	28.9 (0.14)
Having diabetes	Yes	550	8.4
	No	4045	89.7
	Borderline	94	1.9
Kidney Biomarker			
First albumin creatinine ratio		4692	24.1(2.94)
Second albumin creatinine ratio		4692	16.0 (2.06)
First albuminuria	Negative	4183	92.0
	Microalbuminuria	424	6.9
	Macroalbuminuria	85	1.1
Second albuminuria	Negative	4383	95.3
	Microalbuminuria	248	4.0
	Macroalbuminuria	61	0.7
Persistent albuminuria	Negative	4437	96.5
	Positive	255	3.5
eGFR		4692	87.8 (0.74)
eGFR stages	Stage 1	2150	43.2
	Stage 2	2089	49.2
	Stage 3	413	7.1
	Stage 4	29	0.4
	Stage 5	11	0.1
CKD	Negative	4070	90.0
	Positive	622	10.0
CKD levels	Negative	4070	90.0
	Mild	169	2.4
	Moderate-to-Severe	453	7.6
CKD stages	Negative	4070	90.0
	Stage 1	69	0.9
	Stage 2	100	1.5
	Stage 3	413	7.1
	Stage 4	29	0.4
	Stage 5	11	0.1

**Table 2:** Associations between *T. gondii* (positive/negative) and CKD biomarkers, status, and stages.

Variable	Level	*T. gondii* Negative		*T. gondii* Positive		*p*-Value
		*n*	Weighted Percentage or Mean (SE)	*n*	Weighted Percentage or Mean (SE)	
First albumin-to-creatinine ratio		3789	22.5 (3.56)	903	32.9 (4.62)	0.1222
Second albumin-to-creatinine ratio		3789	14.4 (2.38)	903	24.9 (3.91)	0.0376
First albuminuria	Negative	3389	85.1	794	14.9	0.0752
	Microalbuminuria	336	83.5	88	16.5	
	Macroalbuminuria	64	74.1	21	25.9	
Second albuminuria	Negative	3555	85.2	828	14.8	0.0005
	Microalbuminuria	192	78.2	56	21.8	
	Macroalbuminuria	42	67.8	19	32.2	
Persistent albuminuria	Negative	3594	76.9	843	23.1	<0.0001
	Positive	195	85.1	60	14.9	
eGFR		3789	88.1 (0.76)	903	86.2 (1.22)	0.0913
eGFR stages	Stage 1	1758	85.2	392	14.8	0.0014
	Stage 2	1688	85.6	401	14.4	
	Stage 3	317	78.7	96	21.3	
	Stage 4	20	71.7	9	28.3	
	Stage 5	6	68.8	5	31.2	
CKD	Negative	3314	85.6	756	14.4	0.0001
	Positive	475	77.9	147	22.1	
CKD levels	Negative	3314	85.6	756	14.4	<0.0001
	Mild	132	76.9	37	23.1	
	Moderate-to-Severe	343	78.2	110	21.8	
CKD stages	Negative	3314	85.6	756	14.4	0.0004
	Stage 1	58	79.8	11	20.2	
	Stage 2	74	75.1	26	24.9	
	Stage 3	317	78.7	96	21.3	
	Stage 4	20	71.1	9	28.9	
	Stage 5	6	68.8	5	31.2	

**Table 3:** Association between *T. gondii* exposure status and CKD status stratified by age.

Toxoplasma IgG Antibody	Younger (<45 Year)			Older (≥45 Year)		
	No CKD	CKD	Total	No CKD	CKD	Total
Negative (n)	1505	43	1548	1809	432	2241
Positive (n)	239	5	244	517	142	659
Stratified risk ratio	RR_young_ = 0.76 (95% CI: 0.33–1.76)			RR_old_ = 1.11 (95% CI: 0.95–1.31)		
Unstratified risk ratio (RR_crude_) = 1.27 (95% CI: 1.09–1.49)						
Pooled estimate risk ratio (RR_MH_) = 1.09 (95% CI: 0.93–1.28)						
Magnitude of Confounding = 17%						

**Table 4:** Binomial Logistic regression model for CKD status on *T. gondii* IgG antibody (positive/negative) adjusted for the demographic factors and BMI.

	CKD			
Sample Size = 4659	Odds Ratio	OR 95% Confidence Interval		*p*-Value
Toxoplasma IgG antibody (Positive)	1.40	1.06	1.84	0.0447
Age				
Younger (<45) (reference)				
Older (≥45)	8.89	6.31	12.51	<0.0001
Gender				
Male (reference)				
Female	1.27	1.06	1.54	0.0352
Race/ethnicity				
Mexican American (reference)				
Other Hispanic	0.91	0.62	1.34	0.6524
Non-Hispanic White	1.09	0.76	1.55	0.6649
Non-Hispanic Black	0.93	0.59	1.49	0.7786
Other Race	0.90	0.42	1.95	0.8028
BMI	1.04	1.02	1.06	0.0059
Constant	0.01	0.00	0.01	<0.0001
AIC = 2697, Pseudo R-Square = 0.07				

**Table 5:** Binomial Logistic regression model for CKD status on *T. gondii* IgG antibody (positive/negative) adjusted for the demographic factors and other covariates.

	CKD			
Sample Size = 3722	Odds Ratio	OR 95% Confidence Interval		*p*-Value
Toxoplasma IgG antibody (Positive)	1.27	0.97	1.64	0.1381
Age				
Younger (<45) (reference)				
Older (≥45)	8.59	5.48	13.47	0.0002
Gender				
Male (reference)				
Female	1.15	0.93	1.42	0.2487
Race/ethnicity				
Mexican American (reference)				
Other Hispanic	1.00	0.60	1.64	0.9873
Non-Hispanic White	1.19	0.88	1.62	0.2985
Non-Hispanic Black	0.95	0.57	1.55	0.8334
Other Race	0.79	0.35	1.79	0.5932
Alcohol use (No)	0.94	0.71	1.25	0.7102
BMI	1.04	1.01	1.06	0.0144
Has Diabetes (No)	0.40	0.27	0.61	0.0075
Constant	0.03	0.01	0.08	0.0009
AIC = 2120, Pseudo R-Square = 0.08				

**Table 6:** Binomial Logistic regression model for CKD status on *T. gondii* IgG antibody (positive/negative) adjusted for the demographic factors (except age) and other covariates.

	CKD			
Sample Size = 3722	Odds Ratio	OR 95% Confidence Interval		*p*-Value
Toxoplasma IgG antibody (Positive)	1.56	1.20	2.04	0.0168
Gender				
Male (reference)				
Female	1.18	0.98	1.43	0.1464
Race/ethnicity				
Mexican American (reference)				
Other Hispanic	0.97	0.59	1.63	0.9338
Non-Hispanic White	1.70	1.21	2.27	0.0196
Non-Hispanic Black	1.11	0.68	1.82	0.6845
Other Race	0.81	0.39	1.73	0.6046
Alcohol use (No)	0.89	0.67	1.17	0.4407
BMI	1.03	1.01	1.05	0.0219
Has Diabetes (No)	0.30	0.19	0.43	0.0078
Constant	0.24	0.10	0.54	0.0142
AIC = 2299, Pseudo R-Square = 0.03				

**Table 7:** Binomial Logistic regression model for CKD status on *T. gondii* IgG antibody (positive/negative) adjusted for the demographic factors (except age) and other covariates, including smoking.

	CKD			
Sample Size = 1885	Odds Ratio	OR 95% Confidence Interval		*p*-Value
Toxoplasma IgG antibody (Positive)	1.81	1.26	2.60	0.0325
Gender				
Male (reference)				
Female	0.99	0.73	1.33	0.9525
Race/ethnicity				
Mexican American (reference)				
Other Hispanic	0.82	0.38	1.75	0.6348
Non-Hispanic White	1.46	0.88	2.44	0.2133
Non-Hispanic Black	1.45	0.83	2.56	0.2593
Other Race	0.53	0.17	1.68	0.3423
Alcohol use (No)	0.89	0.64	1.27	0.5732
BMI	1.02	1.01	1.04	0.0696
Has Diabetes (No)	0.30	0.19	0.51	0.0125
Smoking use (No) Reference				
Smoking use (every day)	0.93	0.49	1.76	0.8512
Smoking use (some days)	1.78	1.32	2.39	0.0187
Constant	0.24	0.10	0.54	0.1556
AIC = 1142, Pseudo R-Square = 0.04				

**Table 8:** Binomial Logistic regression model for CKD status on *T. gondii* IgG antibody (positive/negative) adjusted for the demographic factors (except age) and other covariates, including drug use.

	CKD			
Sample Size = 1551	Odds Ratio	OR 95% Confidence Interval		*p*-Value
Toxoplasma IgG antibody (Positive)	0.75	0.36	1.56	0.5803
Gender				
Male (reference)				
Female	0.98	0.59	1.64	0.9589
Race/ethnicity				
Mexican American (reference)				
Other Hispanic	1.42	0.76	2.66	0.4678
Non-Hispanic White	0.89	0.55	1.43	0.7176
Non-Hispanic Black	1.12	0.61	2.06	0.7702
Other Race	0.76	0.16	3.60	0.7890
Alcohol use (No)	0.55	0.29	1.06	0.3265
BMI	1.06	1.03	1.09	0.1617
Marijuana/hashish use	1.53	0.90	2.59	0.3589
Cocaine/heroin/methamphetamine use	0.74	0.41	1.34	0.0562
Constant	0.01	0.00	0.02	0.0676
AIC = 416, Pseudo R-Square = 0.01				

**Table 9:** Ordinal Logistic regression model for CKD stages on *T. gondii* IgG antibody (positive/negative) adjusted for the demographic factors and BMI.

	CKD Stages			
Sample Size = 4659	Odds Ratio	OR 95% Confidence Interval		*p*-Value
Toxoplasma IgG antibody (Positive)	1.41	1.07	1.86	0.0424
Age				
Younger (<45) (reference)				
Older (≥45)	8.97	6.38	12.60	<0.0001
Gender				
Male (reference)				
Female	1.32	1.10	1.60	0.0195
Race/ethnicity				
Mexican American (reference)				
Other Hispanic	0.95	0.65	1.40	0.8087
Non-Hispanic White	1.14	0.81	1.60	0.4615
Non-Hispanic Black	0.96	0.61	1.51	0.8650
Other Race	0.91	0.43	1.94	0.8250
BMI	1.04	1.01	1.06	0.0058
Negative|Stage 1	0.13	0.02	0.69	0.0742
Stage 1|Stage 2	0.15	0.08	0.26	0.0852
Stage 2|Stage 3	0.18	0.13	0.27	0.1131
Stage 3|Stage 4	3.68	3.13	4.32	0.2157
Stage 4|Stage 5	18.25	12.39	26.86	0.0223

**Table 10:** Ordinal Logistic regression model for CKD levels on *T. gondii* IgG antibody (positive/negative) adjusted for the demographic factors and BMI.

	CKD Levels			
Sample Size = 4659	Odds Ratio	OR 95% Confidence Interval		*p*-Value
Toxoplasma IgG antibody (Positive)	1.23	0.93	1.63	0.1799
Age				
Younger (<45) (reference)				
Older (≥45)	2.94	2.17	3.97	<0.0001
Gender				
Male (reference)				
Female	1.52	1.25	1.84	0.0027
Race/ethnicity				
Mexican American (reference)				
Other Hispanic	1.50	1.11	2.03	0.0297
Non-Hispanic White	2.25	1.79	2.83	0.0001
Non-Hispanic Black	0.96	0.61	1.51	0.8650
Other Race	1.53	1.10	2.12	0.8250
BMI	1.01	0.99	1.02	0.6051
Mild|Negative	0.10	0.06	0.16	0.0001
Negative|Moderate-to-Severe	76.14	70.78	81.90	0.0001

**Table 11:** Ordinal Logistic regression model for CKD levels on *T. gondii* IgG antibody (positive/negative) adjusted for the demographic factors (except age) and BMI.

	CKD Levels			
Sample Size = 4659	Odds Ratio	OR 95% Confidence Interval		*p*-Value
Toxoplasma IgG antibody (Positive)	1.42	1.07	1.90	0.0371
Gender				
Male (reference)				
Female	1.54	1.28	1.85	0.0011
Race/ethnicity				
Mexican American (reference)				
Other Hispanic	1.50	1.07	2.04	0.0417
Non-Hispanic White	2.78	2.16	3.58	0.0001
Non-Hispanic Black	1.34	0.86	2.12	0.2245
Other Race	1.53	1.10	2.12	0.8250
BMI	1.01	0.99	1.02	0.2861
Mild|Negative	0.08	0.05	0.14	0.0001
Negative|Moderate-to-Severe	50.53	47.75	53.48	0.0001

## Data Availability

The NHANES dataset is publicly available online, accessible at https://www.cdc.gov/nchs/nhanes/index.htm (accessed on 5 January 2022).
